# A Systematic Review of the Efficacy and Safety of Anti-amyloid Monoclonal Antibodies in Alzheimer's Disease

**DOI:** 10.7759/cureus.85377

**Published:** 2025-06-04

**Authors:** Umesh C Chundu, Sree Ram Thiriveedhi, Chetanya Bhatti, Jaya Sai Mupparaju, Nina Otinashvili, Elene Gelishvili

**Affiliations:** 1 Neurology, Guntur Medical College, Guntur, IND; 2 Community Medicine, Katuri Medical College and Hospital, Guntur, IND; 3 Family Medicine, Navjeevan Hospital Hisar, New Delhi, IND; 4 Internal Medicine, NRI Medical College, Vijayawada, IND; 5 Medicine, Tbilisi State Medical University, Tbilisi, GEO

**Keywords:** alzheimer’s disease, amyloid beta protein, amyloid-related imaging abnormalities, anti-amyloid-β monoclonal antibodies, cognition disorders

## Abstract

Monoclonal antibody (mAb) therapies targeting amyloid-beta (Aβ) plaques have gained prominence over the past decade as potential disease-modifying treatments for Alzheimer’s disease (AD), leading to major regulatory approvals and global debate. Nonetheless, the central question persists: does this emerging therapy have a justified role in the treatment protocol for AD? This systematic review evaluates the efficacy and safety of these agents across phase II and III clinical trials conducted in the past decade (2014-2024), aligning with the timeline when disease-modifying therapies gained momentum. A systematic search was performed across PubMed and the Cochrane Library to identify phase II and III randomized controlled trials (RCTs) conducted between January 2014 and December 2024. The inclusion criteria focused on studies that evaluated cognitive outcomes using scales such as the Clinical Dementia Rating-Sum of Boxes (CDR-SB), Alzheimer's Disease Assessment Scale-Cognitive Subscale (ADAS-Cog), and Mini-Mental State Examination (MMSE). Additionally, trials assessing biomarkers, including CSF measures and PET imaging, were included. Safety outcomes, particularly amyloid-related imaging abnormalities (ARIA), were also systematically analyzed. Due to heterogeneity in outcome measures, a narrative synthesis was conducted. Sixteen RCTs met the inclusion criteria. Lecanemab reduced amyloid burden with 81% of patients achieving amyloid-negative PET scans (Clarity AD trial) and showed a 27% reduction in cognitive decline on the ADCOMS scale. While statistically significant, the 27% ADCOMS (Alzheimer's Disease Composite Score) reduction warrants comparison to MCID (minimal clinically important difference) thresholds (e.g., ~0.5-1.0 points for CDR-SB in early AD) to assess real-world impact. Donanemab demonstrated 76% plaque clearance and a 35% slowing in cognitive decline in early AD patients. Aducanumab showed dose-dependent effects on plaque clearance but had inconsistent cognitive outcomes and higher ARIA rates. Recent mAb trials provide promising evidence for disease modification in early AD stages, particularly with lecanemab and donanemab. However, variability in cognitive outcomes and safety concerns warrant cautious interpretation and long-term validation.

## Introduction and background

Alzheimer’s disease

Alzheimer’s disease (AD) is a progressive neurodegenerative disorder characterized by memory loss, cognitive impairment, and a decline in daily functioning. It is the most common cause of dementia, accounting for 60-70% of cases worldwide, and affects over 50 million individuals [1]. While AD primarily manifests in older adults, early-onset cases can occur as early as the third decade of life. According to the Alzheimer’s Association, the financial burden of AD is substantial, with annual care costs exceeding $50,000 per patient, while the emotional toll on caregivers is immeasurable [1]. Projections estimate that the number of individuals living with dementia, including AD, will rise significantly by 2050. This anticipated increase underscores the urgent need for effective, scalable interventions to manage and potentially modify the course of the disease [1]. Despite ongoing research, existing therapies remain largely symptomatic, highlighting an urgent unmet need for disease-modifying treatments. AD is characterized by insidious episodic memory loss and progressive cognitive decline; AD remains a significant global health challenge requiring innovative therapeutic strategies. Moreover, the lack of curative options and the progressive nature of the disease underscore the clinical urgency for effective disease-modifying therapies that can delay or prevent irreversible cognitive decline.

Amyloid hypothesis

The amyloid cascade hypothesis postulates that Aβ accumulation is the primary pathogenic event in AD, setting off a cascade of tau pathology, neuroinflammation, and neuronal loss [2]. This hypothesis has been the foundation for therapeutic strategies aimed at mitigating Aβ pathology [3]. Among these, passive immunization with monoclonal antibodies (mAbs) targeting Aβ has garnered substantial attention due to its potential to enhance Aβ clearance and modify disease progression. Unlike small-molecule inhibitors of β- and γ-secretases, which aim to reduce Aβ production, mAbs facilitate clearance via microglial activation, leading to the phagocytosis and lysosomal degradation of aggregated plaques.

Rationale for the use of anti-monoclonal antibodies

Current treatments for AD include donepezil, rivastigmine, galantamine, and memantine, all of which are approved by the U.S. Food and Drug Administration (FDA) for the treatment of AD. Neither cholinesterase inhibitors nor memantine has proven efficacious in patients with AD. Current pharmacologic treatments for AD, including cholinesterase inhibitors such as donepezil, rivastigmine, and galantamine, as well as the NMDA receptor antagonist memantine, are approved by the U.S. FDA. However, these agents have shown only modest symptomatic benefit and have not demonstrated significant efficacy in altering disease progression. This therapeutic gap has driven the development of novel disease-modifying strategies, most notably mAbs targeting amyloid-beta (Aβ) pathology [1].

Anti-amyloid mAbs have advanced significantly in clinical development, with passive immunization emerging as a promising strategy due to their selectivity and tolerability [4]. These mAbs reduce Aβ plaques by activating microglia, leading to phagocytosis and degradation of fibrillar Aβ via the endosomal-lysosomal pathway. Each approved mAb targets distinct Aβ species, contributing to variations in efficacy and safety profiles. This approach remains central to AD therapeutic research [4]. However, despite regulatory milestones, the field faces substantial debate due to inconsistent trial outcomes and limited understanding of clinical significance. However, despite regulatory milestones, the field is marked by controversy and uncertainty stemming from conflicting clinical trial outcomes. Pivotal trials such as EMERGE and ENGAGE (aducanumab), Clarity AD (lecanemab), and TRAILBLAZER-ALZ (donanemab) have brought these therapies into the spotlight [5-8]. Aducanumab and lecanemab received accelerated FDA approval, based on their demonstrated ability to reduce amyloid burden, while donanemab is currently under regulatory review. The clinical relevance of these approvals remains debated, particularly in light of aducanumab’s approval following one positive trial (EMERGE) and one negative (ENGAGE). This discrepancy highlights the complexity of interpreting amyloid clearance in relation to cognitive outcomes and the need for rigorous post-marketing surveillance. Yet, cognitive outcomes have varied considerably, and adverse events such as amyloid-related imaging abnormalities (ARIA) remain a concern. For example, a Clinical Dementia Rating-Sum of Boxes (CDR-SB) difference of 0.45-0.50 points over 18 months, though statistically significant, may not translate into meaningful functional benefit for patients unless aligned with MCID (minimal clinically important difference) thresholds. In contrast, other mAbs such as bapineuzumab, crenezumab, gantenerumab, and solanezumab have not met efficacy endpoints and remain under investigation.

Research gap and objective

Given the heterogeneity in trial design, outcomes, and safety profiles, there is a critical need to synthesize current evidence and evaluate the therapeutic potential of these agents. Furthermore, a lack of standardized trial designs, variations in population subtypes (e.g., APOE ε4 carriers), and inconsistent endpoints complicate cross-trial comparisons. However, the key question remains: should these therapies be incorporated into the treatment protocol for AD? If so, which mAb offers the most effective and safest option? This review aims to address these questions by critically evaluating the efficacy, safety, and mechanistic rationale of anti-amyloid mAbs, contextualized within biomarker endpoints, clinical relevance, and trial quality.

## Review

Objective

The aim of this review is to evaluate the efficacy and safety of anti-amyloid mAbs in the treatment of AD.

Methodology

Eligibility Criteria

Peer-reviewed primary studies available online were included, guided by the PICO-TS (Population, Intervention, Control, Outcome, Timing, Study design) criteria.

Inclusion Criteria 

This systematic review included all patients diagnosed with AD, regardless of race, gender, or age (Population). The intervention of interest was anti-amyloid mAbs, regardless of dose, route, or frequency of administration (Intervention). Studies were eligible if they included a control group, either through randomized controlled trials (RCTs) or before-and-after comparisons (Control). The primary outcomes assessed were changes in cognitive function measured using the CDR-SB, Alzheimer’s Disease Assessment Scale-Cognitive Subscale (ADAS-Cog), Mini-Mental State Examination (MMSE), and Alzheimer’s Disease Cooperative Study-Activities of Daily Living (ADCS-ADL) (Outcome). Secondary outcomes included biomarker changes such as p181-tau, Aβ42, and Aβ40 in CSF and blood, Aβ plaque burden on PET scans, and the incidence of ARIA (Outcome). Only studies conducted between January 1, 2014, and December 1, 2024, were included (Timing). Eligible publications were restricted to randomized clinical trials in phase II and phase III, published in English, and available as full-text, open-access articles (Study design).

Exclusion Criteria 

Studies were excluded if they involved patients with dementias other than AD (Population), utilized anti-tau antibody therapies (Intervention), or compared one anti-mAb to another instead of a different drug class or placebo (Control). Trials were also excluded if they did not report changes in cognitive function or Aβ plaque load on PET scans (Outcome), were conducted outside January 1, 2014, to December 1, 2024, window (Timing), or if they were observational studies, non-human studies, case reports, phase I trials, grey literature, or incomplete studies (Study design).

The detailed search strategy, including the combination of keywords and MeSH terms, is summarized in Table 3 (see Appendices).

Study Selection

**Figure 1:**
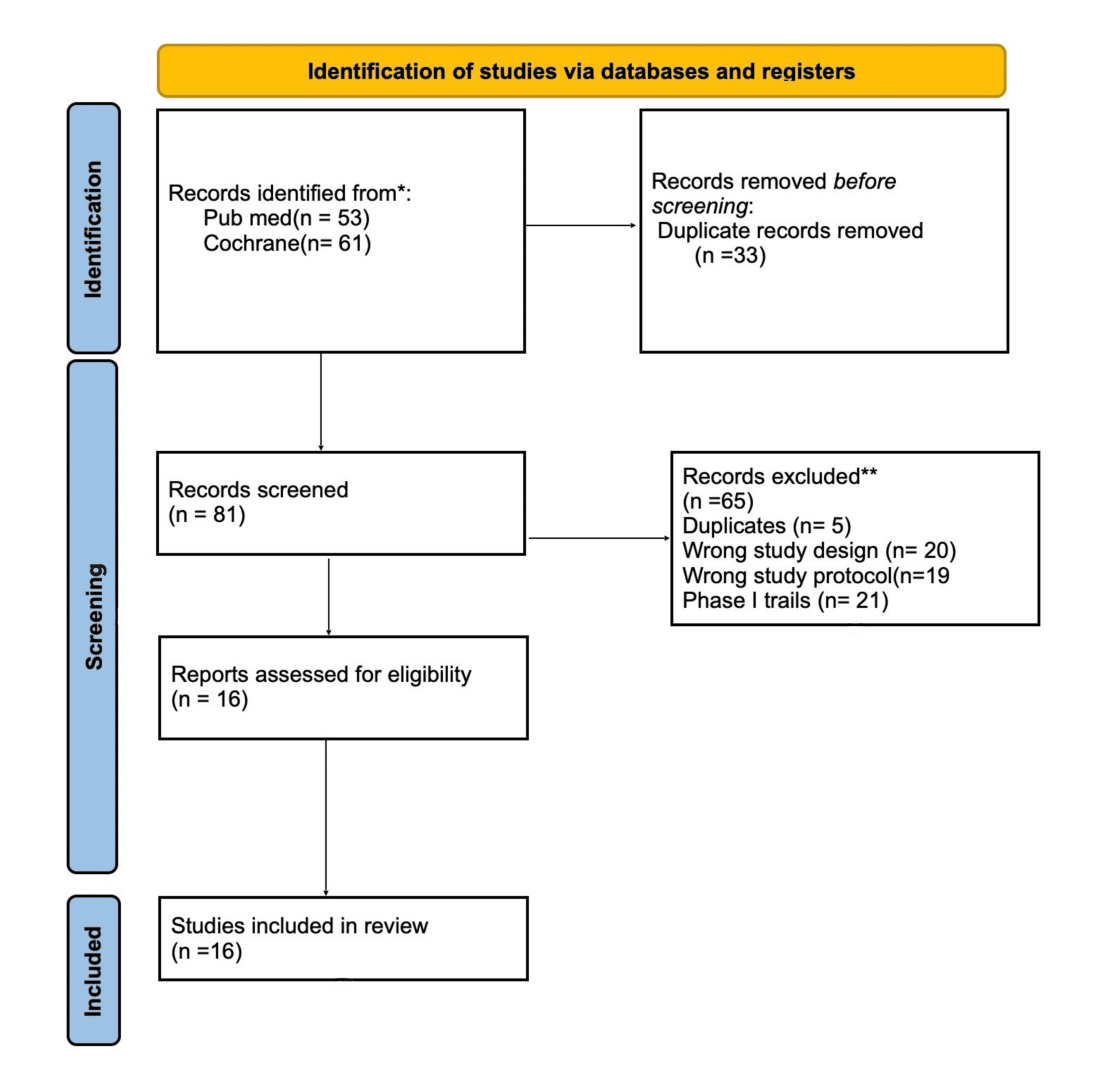
Prisma Flowchart (Study Selection Process) PRISMA, Preferred Reporting Items for Systematic Reviews and Meta-Analyses

Study screening and selection were independently conducted by six reviewers (UC, SR, CB, JM, NO, and EG). Initial screening was performed using titles and abstracts, followed by full-text evaluation based on the inclusion and exclusion criteria to ensure objectivity and minimize selection bias. A total of 114 studies were retrieved in the initial search. After removing 33 duplicates, 81 articles remained. Of these, 63 studies were excluded for not meeting eligibility criteria, and the remaining 16 studies were included in the final analysis. Disagreements during screening were resolved through group discussion and consensus. The complete selection process is illustrated in Figure 1 [9].

Data Extraction

Data from the 16 selected studies were independently extracted by six reviewers (UC, SR, CB, JM, NO, and EG). Extracted data included study characteristics such as first author, publication year, study design, and sample size, independent of drug dosage and duration. Efficacy outcomes included primary cognitive measures such as the CDR-SB, and secondary cognitive assessments including the Alzheimer's Disease Assessment Scale-Cognitive Subscale (ADAS-Cog), Mini-Mental State Examination (MMSE), and the Alzheimer's Disease Cooperative Study-Activities of Daily Living (ADCS-ADL). Secondary outcomes also encompassed amyloid biomarker reductions, specifically p181-tau, Aβ42, and Aβ40, measured in CSF, blood, and PET imaging. Safety outcomes were assessed based on the incidence of ARIA-E (amyloid-related imaging abnormalities-edema), ARIA-H (amyloid-related imaging abnormalities-hemorrhage), and all-cause mortality.

Although inter-reviewer reliability was high, a kappa statistic was not calculated. Discrepancies during data extraction were resolved through consensus meetings.

Quality Assessment - Risk of Bias

The quality of the included research was assessed using the RoB 2 version of the Cochrane Risk of Bias tool [10] to evaluate the risk of bias in RCTs. Based on this assessment, studies were categorized as having poor, fair, or good quality, as shown in Table 1.

**Table 1:** Cochrane Risk of Bias Tool 2.0 This table presents the risk of bias assessment for each included randomized controlled trial, evaluated using the Cochrane Risk of Bias 2.0 (RoB 2.0) tool. The five assessed domains are as follows: Randomization Process (adequacy of random allocation), Deviations from Intended Interventions (compliance and blinding), Missing Outcome Data (completeness and handling of missing data), Measurement of the Outcome (objectivity and consistency in outcome assessment), and Selection of the Reported Result (risk of selective outcome reporting). Each domain is rated as Low Risk, Some Concerns, or High Risk, which together determine the Overall Risk of Bias rating. Most studies were classified as low risk, indicating high methodological quality. However, a few studies were rated as high risk or raised some concerns, primarily due to incomplete data or potential measurement bias. These results support the overall credibility of the included evidence but warrant cautious interpretation of findings from studies with a higher risk of bias.

S. No.	First Author (et al.)	Randomization Process	Deviations From Interventions	Missing Outcome Data	Measurement of Outcomes	Selection of Reported Result	Overall Risk of Bias
1	Budd Haeberlein S, et al. [5]	Unclear risk	Low risk	Some concerns	Low risk	Low risk	Some concern for bias
2	Swanson CJ, et al. [6]	Low risk	Some concerns	High risk	High risk	High risk	High risk
3	van Dyck CH, et al. [7]	unclear risk	High risk	High risk	High risk	High risk	High risk
4	Salloway S, et al. [11]	Low risk	Low risk	Low risk	Low risk	Low risk	Low risk
5	Ivanoiu A, et al. [12]	Low risk	Low risk	Low risk	Low risk	Low risk	Low risk
6	Kettera N, et al. [13]	Low risk	Low risk	Low risk	Low risk	Low risk	Low risk
7	Salloway SP, et al. [14]	Low risk	Low risk	Low risk	Low risk	Low risk	Low risk
8	Vandenberghe R, et al. [15]	Low risk	Low risk	Low risk	Low risk	Low risk	Low risk
9	Brody M, et al. [16]	Low risk	Low risk	Low risk	Low risk	Low risk	Low risk
10	Ostrowitzki S, et al. [17]	Low risk	Some concerns	High risk	Low risk	Low risk	High risk
11	Sims JR, et al. [18]	Low risk	Low risk	Low risk	Low risk	Low risk	Low risk
12	Gueorguieva I, et al. [19]	Low risk	Low risk	Low risk	Low risk	Low risk	Low risk
13	Bateman RJ, et al. [20]	Low risk	Low risk	Low risk	Low risk	Low risk	Low risk
14	McDade E, et al. [21]	Some concerns	Low risk	Some concerns	Some concerns	Low risk	Some concern for bias
15	Honig LS, et al. [22]	Low risk	Low risk	Low risk	Low risk	Low risk	Low risk
16	Doody RS, et al. [23]	Low risk	Low risk	Low risk	Low risk	Low risk	Low risk

Results

A total of 16 RCTs were included in this review, evaluating the efficacy and safety of mAbs in patients with AD. These trials included two studies on Aducanumab [5,11], five on bapineuzumab [12-16], one on crenezumab [17], two on donanemab [18,19], one on gantenerumab [20], three on lecanemab [6,7,21], and two on solanezumab [22,23]. The included studies spanned multiple phases (II and III) and patient populations, with varying durations and inclusion criteria, summarized in Table 2.

**Table 2:** Characteristics of the Studies

S.No.	Author	Year	Drug Used	Study Design	Location	Follow-Up Duration
1	Budd Haeberlein S, et al. [5]	2022	Aducanumab	Phase 3 randomized clinical trial	Multinational	78 weeks
2	Swanson CJ, et al. [6]	2021	Lecanemab	Phase 2b randomized clinical trial	Multinational	18 months
3	van Dyck CH, et al. [7]	2023	Lecanemab	Phase 3 randomized clinical trial	Multinational	18 months
4	Salloway S, et al. [11]	2022	Aducanumab	Phase 3 randomized clinical trial	Multinational	78 weeks
5	Ivanoiu A, et al. [12]	2016	Bapineuzumab	Phase 3 randomized clinical trial	Multinational	Up to 3 years
6	Kettera N, et al. [13]	2017	Bapineuzumab	Phase 3 randomized clinical trial	Multinational	Not specified
7	Salloway SP, et al. [14]	2018	Bapineuzumab	Phase 3 randomized clinical trial	Multinational	3 years
8	Vandenberghe R, et al. [15]	2016	Bapineuzumab	Phase 3 Randomized clinical trial	Global	18 months
9	Brody M, et al. [16]	2016	Bapineuzumab	Phase 2 randomized clinical trial	USA	1 year
10	Ostrowitzki S, et al. [17]	2022	Crenezumab	Phase 3 randomized clinical trial	Multinational	Up to 105 weeks
11	Sims JR, et al. [18]	2023	Donanemab	Phase 3 randomized clinical trial	Multinational	76 weeks
12	Gueorguieva I, et al. [19]	2023	Donanemab	Phase 2 randomized clinical trial	Multinational	52 weeks
13	Bateman RJ, et al. [20]	2023	Gantenerumab	Phase 3 randomized clinical trial	Multinational	116 weeks
14	McDade E, et al. [21]	2022	Lecanemab	Phase 2 randomized clinical trial	Multinational	Average of 24 months
15	Honig LS, et al. [22]	2018	Solanezumab	Phase 3 randomized clinical trial	Multinational	80 weeks
16	Doody RS, et al. [23]	2014	Solanezumab	Phase 3 randomized clinical trial	Multinational	18 months

Primary Efficacy Outcomes

The primary outcome in most studies was a change in cognitive decline, measured by the CDR-SB score. Aducanumab demonstrated a statistically significant reduction in cognitive decline in the EMERGE trial, with a 22% reduction in CDR-SB (P = 0.012) [5]. However, the ENGAGE trial did not replicate these findings, which may reflect heterogeneity in trial populations (e.g., baseline amyloid load or APOE ε4 prevalence), underscoring the need for biomarkers to predict treatment response and explaining the conflicting efficacy data. Lecanemab showed more consistent benefit, with 81% of patients achieving amyloid-negative conversion, and a 25-31% decline in cognitive function over 18 months (P < 0.001) [7]. Similarly, donanemab demonstrated a significant slowing of cognitive decline in early symptomatic AD, with a reduction in CDR-SB scores and improved patient outcomes [18,19].

On the other hand, bapineuzumab showed no significant cognitive benefits in the trials reviewed [12], while gantenerumab and solanezumab also failed to achieve meaningful reductions in CDR-SB scores. In the GRADUATE I trial, gantenerumab showed a mean difference of -0.31 (95% CI, -0.66 to 0.05; P = 0.10), and in GRADUATE II, the difference was -0.19 (95% CI, -0.55 to 0.17; P = 0.30) [20]. Likewise, solanezumab had no significant effect on cognitive decline, with a reported mean difference of 0.1 (95% CI, -0.3 to 0.6; P = 0.51) in EXPEDITION 1 and -0.3 (95% CI, -0.7 to 0.2; P = 0.17) in EXPEDITION 2 [22,23].

Lecanemab also showed small but significant improvements in other cognitive measures such as the ADAS-Cog (P < 0.001), while aducanumab and donanemab provided modest cognitive improvements in their respective trials [7]. In contrast, crenezumab, solanezumab, and bapineuzumab did not demonstrate any significant improvements in cognitive performance [7,12,17,22]. Similarly, Donanemab and lecanemab demonstrated small but significant improvements in MMSE (P < 0.001), while other therapies did not achieve similar benefits [5,7,18].

Additionally, lecanemab and donanemab showed substantial improvements in ADCS-ADL (P < 0.001), indicating a positive effect on functional outcomes, while other treatments showed only minimal functional benefits.

Secondary Outcomes - Amyloid Biomarker Reduction

Lecanemab achieved 81% amyloid-negative conversion, correlating with cognitive benefits [7]. Donanemab also significantly reduced amyloid burden, evidenced by PET and CSF analyses [18]. Bapineuzumab showed minimal amyloid clearance without clinical benefit [12]. Aducanumab showed dose- and time-dependent amyloid reductions, but clinical outcomes were inconsistent [5].

Crenezumab did not show significant reductions in either blood or CSF amyloid levels (P = 0.41), though it did demonstrate some level of biomarker engagement [17]. Similarly, solanezumab and gantenerumab exhibited insignificant effects on amyloid plaque clearance, further confirming their limited efficacy in this regard [20,22].

Safety Outcomes

ARIA is a primary concern in trials involving mAbs targeting amyloid. Aducanumab showed the highest incidence of ARIA-E, with a prevalence of 35.2% in the EMERGE trial [11]. The occurrence of ARIA-E was higher in APOE ε4 carriers, leading to increased discontinuation rates and symptoms such as headaches, dizziness, and confusion. Donanemab also exhibited a high ARIA-E incidence (27.5%), with a notable increase in risk among APOE ε4 carriers (24%) [18].

Lecanemab had lower ARIA-E incidence (12-14%), and most cases were asymptomatic, which were manageable with treatment adjustments [7]. Gantenerumab exhibited ARIA-E in 24.9% of patients, with the highest occurrence in APOE ε4 homozygous carriers (47.8%) [20]. Bapineuzumab had a moderate risk of ARIA-E, with rates ranging from 11% to 21% in APOE ε4 carriers and 4% to 11% in non-carriers [14]. Both crenezumab and solanezumab demonstrated the lowest risk of ARIA-E, with no significant cases reported [17,22].

In addition to ARIA-E, ARIA-H was observed most frequently in patients treated with gantenerumab (5% symptomatic cases), followed by aducanumab and donanemab [18-20]. Lecanemab had a lower incidence of ARIA-H [7], and crenezumab and solanezumab posed minimal risk [17,22]. The elevated mortality risk with bapineuzumab (NNH = 102) may reflect severe ARIA events or selection bias in discontinued trials, though attribution remains unclear without post-hoc safety analyses [12].

Heterogeneity Analysis Results

Cochran’s Q was calculated to be 6.73, which reflects the total variability across the included studies. With four degrees of freedom (df = k-1 = 5-1), this value suggests moderate variability; however, it is not statistically significant, as the associated p-value is greater than 0.05. The I² statistic was found to be 40.6%, indicating moderate heterogeneity. This heterogeneity may arise from variability in mAb dosing regimens (e.g., monthly vs. biweekly) or enrollment of mixed AD stages (prodromal vs. mild dementia). This variability was a key reason for not conducting a meta-analysis.

This variability also precluded the summarization of outcomes in a single table, as combining results across heterogeneous studies would reduce interpretability and clinical relevance.

Discussion

Approved Therapies

Aducanumab, donanemab, and lecanemab have garnered approval for therapeutic use in AD. These mAbs specifically target amyloid plaques, a hallmark of AD pathology, showing promise in slowing cognitive decline in early-stage patients. However, their efficacy and safety profiles differ, necessitating a nuanced and comparative approach in clinical application.

Aducanumab demonstrated a significant reduction in amyloid plaques in the high-dose group in the EMERGE trial. Despite this, its clinical benefit was inconsistent, as modest cognitive improvements were observed only in the EMERGE trial but not in ENGAGE. The conflicting results between EMERGE and ENGAGE may reflect heterogeneity in trial populations (e.g., baseline amyloid load or APOE ε4 prevalence), underscoring the need for biomarkers to predict treatment response. The ARIA-E incidence was 35.2% in high-dose patients, particularly among APOE ε4 carriers. Though these symptoms typically resolve over time, the high incidence of ARIA-E calls for cautious use, with close monitoring for potential adverse effects. Thus, while aducanumab can be employed in therapy, it requires vigilant patient monitoring to ensure safety, particularly in those with genetic risk factors like APOE ε4.

Donanemab has shown significant efficacy, particularly in improving cognition and reducing amyloid plaque load. In contrast to aducanumab, donanemab's ARIA incidence was lower at 27.5%. The drug demonstrated a robust biomarker effect, suggesting its potential as a disease-modifying treatment. However, its application may also be limited to earlier stages of AD, as the impact on cognition diminishes with increasing neurodegeneration.

Lecanemab, another promising mAb, achieved 81% amyloid-negative conversion after 18 months and reduced cognitive decline by 27% based on the Alzheimer's Disease Composite Score (ADCOMS). Lecanemab’s effect is most pronounced in the early stages of AD when amyloid accumulation drives neurodegeneration. This suggests a narrow therapeutic window for late-stage AD, where other factors such as tau pathology and neuroinflammation become more dominant, limiting the efficacy of amyloid-targeting therapies.

A comparative benefit-risk synthesis highlights that while aducanumab has a higher ARIA risk, lecanemab offers more consistent cognitive benefits with a lower incidence of adverse events. Donanemab’s profile lies between these, with robust efficacy but some safety concerns, underscoring the need for individualized treatment decisions.

Experimental and Emerging Therapies

Several other mAbs have been tested, though their clinical outcomes have been less promising. Crenezumab demonstrated limited efficacy but had a favorable safety profile, with a low incidence of ARIA. It may have utility in patients who cannot tolerate other treatments, but its lack of significant cognitive improvement curtails its therapeutic potential.

Gantenerumab, like other amyloid-targeting therapies, reduced amyloid plaques in about 28% of participants. However, it failed to show significant cognitive benefits, limiting its therapeutic value. Solanezumab showed no significant changes in either clinical outcomes or biomarkers, and its minimal adverse effects are unlikely to make it a viable treatment option.

The limitations of amyloid-targeting therapies highlight the multifactorial nature of AD. While amyloid reduction has been a focal point, it may not be sufficient to address other pathological processes such as tau aggregation, neuroinflammation, and synaptic dysfunction, all of which contribute to AD progression. Therefore, emerging therapies targeting these pathways, such as anti-tau mAbs and anti-inflammatory agents, may complement anti-amyloid treatments and offer additional benefits, particularly in the later stages of AD.

Notably, anti-tau therapies such as semorinemab and zagotenemab have shown limited clinical efficacy, with zagotenemab demonstrating a higher incidence of adverse events that may impede its development [24,25]. These findings underscore the importance of targeting multiple pathological pathways concurrently.

Challenges and Future Suggestions

One significant challenge in the treatment of AD is the APOE ε4 genetic variant, which not only predisposes individuals to a higher amyloid burden but also increases the risk of ARIA. As a result, tailored dosing and rigorous monitoring protocols are essential to minimize adverse effects in APOE ε4 carriers. This necessitates the development of personalized treatment approaches based on genetic profiles, which may help optimize therapeutic efficacy while reducing risks.

The modest clinical improvements observed with amyloid-targeting therapies suggest that addressing other factors influencing cognition, such as tau aggregation, neuroinflammation, and synaptic dysfunction, is crucial. Additionally, the high cost of these therapies and the lack of standardized biomarkers to measure efficacy present significant barriers to treatment accessibility and evaluation. The trials included in this review were conducted for relatively short durations (typically 18 months), and long-term data is essential to better understand the safety and effectiveness of these mAbs in diverse patient populations with varying comorbidities.

Moderate heterogeneity across trials (e.g., differences in dosing regimens and patient disease stage) may also contribute to variable outcomes, warranting cautious interpretation of pooled efficacy.

Therefore, ongoing phase IV trials are vital for confirming the long-term benefits and safety profiles of mAb therapies in real-world settings. Cost-effective strategies that incorporate combination therapies and focus on early intervention will be crucial to overcoming the limitations observed in these trials and ensuring broader patient access to effective treatments. Furthermore, exploring biomarkers to identify the best candidates for treatment and improve the targeting of therapies will be essential for the success of future AD treatment.

Additionally, long-term cost-effectiveness analyses and health-economic assessments must be integrated into future research to guide policy and clinical practice.

Limitations

This systematic review has several limitations that may impact its comprehensiveness and generalizability. First, the review utilized only two databases, which may have resulted in the omission of relevant studies indexed in other major platforms such as Embase, Scopus, or Web of Science.

Second, the inclusion criteria were limited to freely accessible full-text articles, potentially excluding high-quality studies published in subscription-based journals and introducing selection bias. This restriction may also contribute to publication bias, as open-access studies are sometimes more likely to report significant findings.

Third, the exclusion of grey literature may bias efficacy estimates, particularly for agents with terminated trials (e.g., crenezumab’s CREAD program), and should be noted as a limitation. Variations in study designs, population characteristics, drug dosages, and outcome measures among the included studies further add to heterogeneity, complicating the synthesis and interpretation of results. This heterogeneity hinders the generalizability of the review findings in real-world settings.

Fourth, the studies included in the review focused on short-duration follow-ups (six to 18 months), which may not fully assess the long-term outcomes of anti-mAb therapy. The restricted outcome scope (e.g., omission of certain cognitive and functional markers) was due to limited availability across trials. Potential publication bias and industry sponsorship of most included trials must also be considered. Finally, language restrictions may have limited the scope of included studies, potentially excluding relevant evidence published in languages other than English.

## Conclusions

This systematic review of anti-amyloid mAbs found that lecanemab, donanemab, and aducanumab showed significant efficacy in improving cognition and reducing amyloid plaques, particularly in early-stage AD. However, the clinical benefits were modest, and while lecanemab and donanemab demonstrated lower incidences of ARIA compared to aducanumab, safety concerns remain, especially in APOE ε4 carriers. Crenezumab, gantenerumab, and solanezumab showed minimal to no cognitive or biomarker improvements and were associated with higher incidences of ARIA and other adverse effects, limiting their therapeutic value.

Although promising in early-stage AD, these therapies may have limited efficacy in later stages where tau pathology and neuroinflammation dominate. The review underscores the importance of personalized treatment approaches, especially considering the genetic profile of APOE ε4 carriers, who are more susceptible to adverse effects. Long-term, large-scale studies are essential to assess the safety, efficacy, and real-world impact of these therapies, particularly in diverse populations. While anti-amyloid mAbs hold promise, their use must be approached cautiously, and combination therapies may be necessary to address the multifactorial nature of AD.

## Appendices

Search strategy

A comprehensive literature search was conducted using the keywords Alzheimer’s Disease; Anti-Amyloid Monoclonal Antibodies; Efficacy; and Safety and Adverse Effects. Relevant articles were identified through PubMed (utilizing MeSH terms) and the Cochrane Library, covering the period from January 1, 2014, to December 1, 2024. The search was restricted to studies published in the English language.

**Table 3:** Search Strategy for Identification of Studies

Component	Details
Databases Searched	PubMed (MeSH), Cochrane Library
Language Restriction	English
Date Range	January 1, 2014 - December 1, 2024
Keywords Used	Alzheimer’s Disease; Anti-Amyloid Monoclonal Antibodies; Efficacy; Safety and Adverse Effects
Population (Disease)	Keywords: "Mild cognitive impairment" OR "MCI" OR "Alzheimer’s disease" OR "AD" MeSH Terms: "Alzheimer Disease/complications"[Mesh] OR "Alzheimer Disease/drug therapy"[Mesh] OR "Alzheimer Disease/immunology"[Mesh] OR "Alzheimer Disease/pathology"[Mesh] OR "Alzheimer Disease/physiopathology"[Mesh] OR "Alzheimer Disease/prevention and control"[Mesh]
Intervention (Drug)	Keywords: "Anti-amyloid antibody" OR "monoclonal antibody" OR "Aducanumab" OR "Lecanemab" OR "Donanemab" OR "Gantenerumab" OR "Solanezumab" MeSH Terms (Major Topic): "Antibodies, Monoclonal/administration and dosage"[Majr] OR "Antibodies, Monoclonal/adverse effects"[Majr] OR "Antibodies, Monoclonal/biosynthesis"[Majr] OR "Antibodies, Monoclonal/drug effects"[Majr] OR "Antibodies, Monoclonal/poisoning"[Majr] OR "Antibodies, Monoclonal/therapeutic use"[Majr] OR "Antibodies, Monoclonal/toxicity"[Majr]
Efficacy Outcomes	Keywords: "Efficacy" OR "effectiveness" OR "treatment outcome" OR "Cognitive decline" OR "cognitive function" OR "cognition" OR "Amyloid reduction" OR "amyloid PET" OR "cerebrospinal fluid amyloid" OR "Disease progression"
Safety Outcomes	Keywords: "Safety" OR "adverse effects" OR "side effects" OR "Amyloid-related imaging abnormalities" OR "ARIA" OR "cerebral edema" OR "microhaemorrhages" OR "Tolerability" OR "dropout rate" OR "adverse events"

## References

[1] Seeley WW, Miller BL (2020). Alzheimer’s Disease. Harrison's Principles of Internal Medicine.

[2] Sadigh-Eteghad S, Sabermarouf B, Majdi A, Talebi M, Farhoudi M, Mahmoudi J (2015). Amyloid-beta: a crucial factor in Alzheimer's disease. Med Princ Pract.

[3] Luan K, Rosales JL, Lee KY (2013). Viewpoint: crosstalks between neurofibrillary tangles and amyloid plaque formation. Ageing Res Rev.

[4] Panza F, Lozupone M, Logroscino G, Imbimbo BP (2019). A critical appraisal of amyloid-β-targeting therapies for Alzheimer disease. Nat Rev Neurol.

[5] Budd Haeberlein S, Aisen PS, Barkhof F (2022). Two randomized phase 3 studies of aducanumab in early Alzheimer's disease. J Prev Alzheimers Dis.

[6] Swanson CJ, Zhang Y, Dhadda S (2021). A randomized, double-blind, phase 2b proof-of-concept clinical trial in early Alzheimer's disease with lecanemab, an anti-Aβ protofibril antibody. Alzheimers Res Ther.

[7] van Dyck CH, Swanson CJ, Aisen P (2023). Lecanemab in early Alzheimer's disease. N Engl J Med.

[8] Mintun MA, Lo AC, Duggan Evans C (2021). Donanemab in early Alzheimer's disease. N Engl J Med.

[9] Haddaway NR, Page MJ, Pritchard CC, McGuinness LA (2022). Prisma2020: An R package and shiny app for producing Prisma 2020-compliant flow diagrams, with interactivity for optimised digital transparency and open synthesis. Campbell Syst Rev.

[10] (2025). RoB 2: a revised Cochrane risk-of-bias tool for randomized trials | Cochrane Bias. https://methods.cochrane.org/bias/resources/rob-2-revised-cochrane-risk-bias-tool-randomized-trials..

[11] Salloway S, Chalkias S, Barkhof F (2022). Amyloid-related imaging abnormalities in 2 Phase 3 studies evaluating aducanumab in patients with early Alzheimer disease. JAMA Neurol.

[12] Ivanoiu A, Pariente J, Booth K (2016). Long-term safety and tolerability of bapineuzumab in patients with Alzheimer's disease in two phase 3 extension studies. Alzheimers Res Ther.

[13] Ketter N, Brashear HR, Bogert J (2017). Central review of amyloid-related imaging abnormalities in two phase III clinical trials of bapineuzumab in mild-to-moderate Alzheimer's disease patients. J Alzheimers Dis.

[14] Salloway SP, Sperling R, Fox NC (2018). Long-term follow up of patients with mild-to-moderate Alzheimer's disease treated with bapineuzumab in a phase III, open-label, extension study. J Alzheimers Dis.

[15] Vandenberghe R, Rinne JO, Boada M (2016). Bapineuzumab for mild to moderate Alzheimer's disease in two global, randomized, phase 3 trials. Alzheimers Res Ther.

[16] Brody M, Liu E, Di J (2016). A phase II, randomized, double-blind, placebo-controlled study of safety, pharmacokinetics, and biomarker results of subcutaneous bapineuzumab in patients with mild to moderate Alzheimer's disease. J Alzheimers Dis.

[17] Ostrowitzki S, Bittner T, Sink KM (2022). Evaluating the safety and efficacy of crenezumab vs placebo in adults with early Alzheimer disease: two phase 3 randomized placebo-controlled trials. JAMA Neurol.

[18] Sims JR, Zimmer JA, Evans CD (2023). Donanemab in early symptomatic Alzheimer disease: the TRAILBLAZER-ALZ 2 randomized clinical trial. J Am Med Assoc.

[19] Gueorguieva I, Willis BA, Chua L (2023). Donanemab population pharmacokinetics, amyloid plaque reduction, and safety in participants with Alzheimer's disease. Clin Pharmacol Ther.

[20] Bateman RJ, Smith J, Donohue MC (2023). Two phase 3 trials of gantenerumab in early Alzheimer's disease. N Engl J Med.

[21] McDade E, Cummings JL, Dhadda S (2022). Lecanemab in patients with early Alzheimer's disease: detailed results on biomarker, cognitive, and clinical effects from the randomized and open-label extension of the phase 2 proof-of-concept study. Alzheimers Res Ther.

[22] Honig LS, Vellas B, Woodward M (2018). Trial of solanezumab for mild dementia due to Alzheimer's disease. N Engl J Med.

[23] Doody RS, Thomas RG, Farlow M (2014). Phase 3 trials of solanezumab for mild-to-moderate Alzheimer's disease. N Engl J Med.

[24] Teng E, Manser PT, Pickthorn K (2022). Safety and efficacy of semorinemab in individuals with prodromal to mild Alzheimer disease: a randomized clinical trial. JAMA Neurol.

[25] Fleisher AS, Munsie LM, Perahia DG (2024). Assessment of efficacy and safety of Zagotenemab: results from PERISCOPE-ALZ, a Phase 2 study in early symptomatic Alzheimer disease. Neurology.

